# Hardware Accelerated Design of a Dual-Mode Refocusing Algorithm for SAR Imaging Systems

**DOI:** 10.3390/s23042143

**Published:** 2023-02-14

**Authors:** Le Yu, Yaqi Li, Nansong Wu

**Affiliations:** 1 School of Artificial Intelligence, Beijing Technology and Business University, Beijing 100048, China; 2 China Light Industry Key Laboratory of Industrial Internet and Big Data, Beijing 100048, China; 3 Department of Engineering, Sonoma State University, Rohnert Park, CA 94928, USA

**Keywords:** SAR imaging, refocusing, data enhancement, FPGA

## Abstract

The use of satellite synthetic aperture radar (SAR) for moving target imaging has gained popularity recently. Researchers are focused on improving its imaging quality. To achieve high-quality and fast imaging, we have developed a dual-mode refocusing algorithm. We optimized the algorithm’s target speed estimation and carried out data enhancement and quantization design for SAR image refocusing. The design is implemented on a Xilinx XC5VFX130T FPGA. The dual-mode image data are based on a slice size of 512 × 512 for slice mode and 256 × 256 for scan mode in a time-series function simulation. The serial–parallel conversion and pipeline design balances the operating speed and logic resources for optimal performance. Experiment results on slice data of real SAR images show that the system’s processing speed can reach two frames per second, utilizing 69633 LUTs, 255 RAMs, and 296 DSPs.

## 1. Introduction

**Figure 1:**
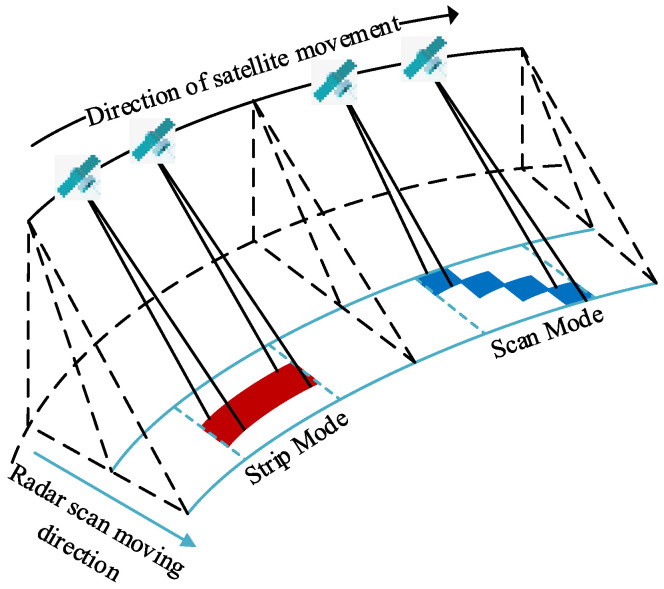
Strip mode and scan mode SAR imaging diagram.

Synthetic aperture radar (SAR) [1,2] is an active microwave remote sensing tool that operates in all weather conditions and can be used at any time of day. It has various applications in the civil field, including terrain mapping, disaster rescue, and urban surveying [3]. The use of SAR for moving target imaging has gained popularity recently. Researchers are focused on improving its imaging quality [4]. SAR images can be obtained through airborne or spaceborne platforms, and SAR can operate in either strip mode [5] or scan mode, depending on the imaging needs. Figure 1 depicts the schematic diagram of the two modes of SAR imaging using a satellite.

In the strip mode, the airborne or spaceborne platform moves, while the antenna remains in a fixed direction. The azimuth resolution is based on the length of the antenna. As the antenna sweeps across the ground at a constant speed, it creates a continuous strip coverage area, highlighted in red in Figure 1. The length of the strip is determined by the distance the radar platform travels. The resulting image is large, so simplifying the subsequent image data refocusing process is necessary to reduce computation time. The strip mode imposes limitations on strip width and requires image data filtering. In the scan mode, the antenna scans along the range direction within a synthetic aperture time, as depicted by the blue area in Figure 1, resulting in a wider imaging area at the cost of azimuthal resolution. The image data obtained in this mode are smaller compared to the strip mode. They use less bandwidth and do not require filtering.

The frequency of the reflected signal and Doppler center frequency change due to the radial movement of the target, causing the SAR image to become blurred. Refocusing, also known as motion compensation, is an important process used to address this issue [6]. Refocusing has two stages: coarse compensation [7] and phase compensation. Phase compensation is crucial in resolving the Doppler frequency shift caused by motion and addressing the shortcomings of coarse compensation.

Phase compensation methods can be divided into two categories. The first category comprises scatter-based algorithms, such as phase gradient autofocus (PGA) [8,9,10], which filters the data using the center offset and window and then estimates the error, but requires a larger window length and longer iteration time [8]. The second category comprises optimization algorithms, such as Doppler centroid tracking (DCT) [11], maximum contrast (MC) [12], maximum likelihood (ML) [13], and minimum entropy (ME) [14]. Although DCT is robust with a low computation cost, it can produce significant errors when imaging multiple moving targets at the same time [15]. Both categories of phase compensation methods require only small sample data, which allows for cascaded end-to-end training and testing, and real-time updates of model parameters to accommodate variations in object size, speed, and orientation. However, both categories suffer from high algorithmic complexity, resulting in low efficiency [16,17]. To address this, field-programmable gate arrays (FPGAs) are often used to accelerate the algorithms.

In recent years, researchers have explored the use of deep neural networks in SAR image refocusing [18,19,20]. Lu et al. used an improved U-net convolutional neural network for refocusing [21]. Image quality was improved in the case of large azimuth velocity, and the image resolution was also improved. Zhou et al. combined the modified real-time recurrent regression (mRe^3^) network with trajectory smoothing long short-term memory (LSTM) for refocusing [22] and was able to reduce the distance error of the target center and fluctuation. However, these methods require a large training dataset, and the model parameters are typically pretrained for FPGA design, lacking the ability for real-time training and testing on SAR images.

To address the crucial issue of refocusing in SAR imaging, especially for both the strip mode and the scan mode, we present a new dual-mode refocusing algorithm for high-quality and fast imaging of moving targets. Our key contributions include:

(1) A novel dual-mode SAR imaging refocusing algorithm that utilizes DCT based on azimuth velocity matching. The algorithm enhances image focus and reduces entropy and contrast.
(2) Lower computational complexity. The optimized speed search for azimuth matching reduces the amount of data by 80%, and the use of binary search further improves search efficiency.
(3) Data enhancement and quantization on the fine refocusing results, compressing data width to 8 bits. The use of a pipeline structure optimizes system performance, balancing speed and resource utilization. The proposed system is implemented on a Xilinx XC5VFX130T FPGA, with simulation results demonstrating two frames per second when processing slice data of real SAR images. The system utilizes significantly less in terms of resources at 69,633 LUTs, 255 RAMs, and 296 DSPs.

The rest of this paper is structured as follows: Section 2 presents the proposed refocusing algorithm. Section 3 details the design and implementation of the refocusing system. Section 4 covers design optimization. Section 5 demonstrates system verification through simulations, and Section 6 concludes the paper and suggests directions for future research.

## 2. SAR Imaging Refocusing Model

**Figure 2:**
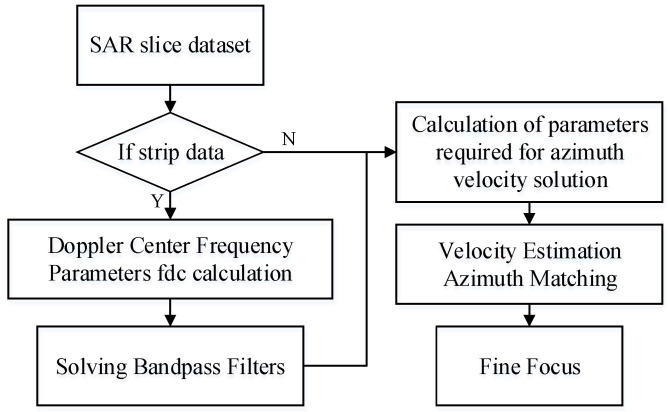
Flowchart of dual-mode SAR imaging refocusing system.

The proposed refocusing method uses a DCT approach based on azimuth velocity to refocus moving targets in SAR images that can be in either strip or scan mode. To meet the bandwidth requirements of the strip mode, a bandpass filter is included in the design. A flowchart of the dual-mode SAR imaging refocusing algorithm is presented in Figure 2.

The proposed algorithm calculates the parameters using sliced data for both the strip and scan modes. The relative motion between the moving target and the observation platform leads to defocusing, hence ’it is crucial to estimate the absolute speed of the moving target. For this, the azimuth velocity must be calculated for both modes. However, the bandwidth limitations of the strip mode require the addition of a bandpass filter constructed from the slice data.

### 2.1. Strip Mode Bandpass Filter

A bandpass filter is required to process the image data in the strip mode due to its bandwidth limitations. The center frequency is calculated using the Doppler frequency. The Doppler center frequency *fdc_est_* is estimated according to the frequency-domain representation of the image data and the pulse frequency of the imaging signal, as shown in Formula (1):

(1) $$fdc_{est}=\left[\left(y-\frac{1}{2}\times NA\right)/NA\right]\times PRF_{up}$$

where *fdc_est_* is the Doppler center frequency; *y* is the index number of the maximum value corresponding to the second-order polynomial fitting of the one-dimensional array obtained by row-wise Fourier transform and column-wise summation; *PRF_up_* is the upper limit of the pulse repetition frequency; and *NA* is the number of columns of the two-dimensional image data.

*Fdc_est_* is not only used as the center frequency of the strip mode window parameter construction, but also needs to be used as the threshold judgment condition for the estimation of the azimuth velocity parameter. According to Formula (2), the effective frequency center point *NC* is calculated by using the Doppler center frequency:

(2) $$NC=\left\lfloor \frac{\left(fdc_{est}-\left\lfloor fdc_{est}/PRF_{up}\right\rfloor \times PRF_{up}\right)\times NA\times PRF_{up}}{2}\right\rfloor +1$$

where *LE* is the strip mode bandwidth correction; and *B* is the effective bandwidth. The bandwidth of the bandpass filter also needs to be calculated according to the upper limit of the pulse repetition frequency, and Taylor estimation is performed according to the bandwidth to form a Taylor standard window. Combining the frequency center point and the Taylor window to construct the bandpass filter required by the strip mode results in the following:

(3) $$LE=2\times \left\lfloor \frac{B\times NA\times PRF_{up}}{2}\right\rfloor$$

### 2.2. Azimuth Velocity

In phase compensation, accurate azimuth velocity is the key to ensuring the quality of compensation. In the azimuth velocity estimation, this paper uses the contrast range azimuth to estimate the velocity approximation. In order to reduce the amount of calculation, the slice data are preferentially filtered. According to Formula (4), the comparison range azimuth is calculated by column, and the larger 20% column data are selected for estimation.

(4) $$cn=\frac{\sqrt{NA\times \sum_{i=1}^{NA}{\left(\left|d_{i}\right|-\frac{1}{NA}\sum_{j=1}^{NA}\left|d_{j}\right|\right)}^{2}}}{\sum_{j=1}^{NA}\left|d_{j}\right|}$$

In the above formula, *i* is an integer, and −*NA*/2 ≤ *i* ≤ *NA*/2 – 1, *d_i_* represents the *i* data in the column; *j* is an integer, and −*NA*/2 ≤ *j* ≤ *NA*/2 – 1, *d_j_* represents the *j* data of the column. According to Formulas (5) and (6), the pre-estimated azimuth velocities are respectively phase-calculated for the scan mode and the strip mode.

(5) $$H_{i}=e^{j\left[\left(4\times \pi \times vc\times V\right)\times WL/RF\right]\cdot ta_{i}{}^{2}}$$

(6) $$H_{i}=e^{j\left[\left(\pi \times vc\times V\times WL\times RF\right)/V^{3}\right]\cdot fa_{i}{}^{2}}$$

In these formulas, *H* is the phase; *vc* is the speed to be estimated; *V* is the platform speed; and *WL* is the waveform length. In the scan mode, the time-series parameter *ta* is calculated according to Formula (7). Unlike the scan mode, the *fa* sequence parameters in the strip mode are calculated according to Equation (8):

(7) $$ta_{i}=\frac{i\times Nu}{PRF_{up}\times NA}$$

(8) $$fa_{j}=\left\{\begin{array}{cc} \frac{PRF_{up}\times (i-1+NA)}{NA} & \begin{array}{c} when\left(fdd\geq PRF_{up}/2\right)\\ and\left(i<\left[\left(fdd-PRF_{up}/2\right)\times NA/PRF_{up}+1\right]\right) \end{array}\\ \frac{PRF_{up}\times (i-1)}{NA} & \begin{array}{c} when\left(fdd\geq PRF_{up}/2\right)\\ and\left(i\geq \left[\left(fdd-PRF_{up}/2\right)\times NA/PRF_{up}+1\right]\right) \end{array}\\ \frac{PRF_{up}\times (i-1+NA)}{NA} & \begin{array}{c} when\left(fdd<PRF_{up}/2\right)\\ and\left(i<\left[\left(fdd+PRF_{up}/2\right)\times NA/PRF_{up}+1\right]\right) \end{array}\\ \frac{PRF_{up}\times (i-1)}{NA} & \begin{array}{c} when\left(fdd<PRF_{up}/2\right)\\ and\left(i\geq \left[\left(fdd+PRF_{up}/2\right)\times NA/PRF_{up}+1\right]\right) \end{array} \end{array}\right.$$

where *fdd* is calculated according to Formula (9).

(9) $$fdd=fdc_{est}-\left\lfloor fdc_{est}/PRF_{up}\right\rfloor \times PRF_{up}$$

When estimating speed, it is necessary to give priority to phase compensation, which is calculated according to Formula (10):

(10) $$da_{i}=F\left[H_{i}\times \left(F^{-1}\left(d_{i}\right)\right)\right]$$

According to Formula (4), the compensation data are brought into the calculation of the contrast range azimuth, and it becomes Formula (11) after substituting:

(11) $$cnr=\frac{\sqrt{NA\times \sum_{i=1}^{NA}{\left[\left|F\left(H_{i}\times F^{-1}\left(d_{i}\right)\right)\right|-\frac{1}{NA}\sum_{j=1}^{NA}\left|F\left(H_{j}\times F^{-1}\left(d_{j}\right)\right)\right|\right]}^{2}}}{\sum_{j=1}^{NA}\left|F\left(H_{j}\times F^{-1}\left(d_{j}\right)\right)\right|}$$

### 2.3. Moving Target Fine Focus

**Figure 3:**
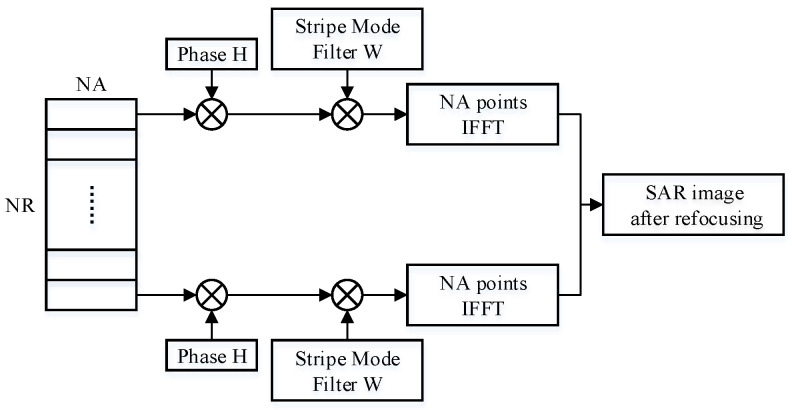
Moving target fine focus model.

The proposed algorithm achieves fine focusing of the moving target through phase compensation, which is determined using the estimation of the azimuth velocity. The slice data are convolved with the corresponding phase data, and the response is complex multiplication on the spectrum. As shown in Figure 3, the strip mode needs to filter the slice data. Therefore, in the frequency domain, multiplication not only with the phase data, but also with the filter is required to achieve windowing in the time domain. The slice data are the frequency-domain complex data after Fourier transform.

**Figure 4:**
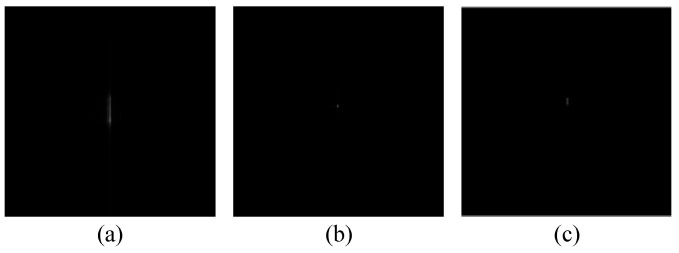
Strip mode moving target imaging: (**a**) image before refocusing; (**b**) image after refocusing; and (**c**) image after deep learning.

**Figure 5:**
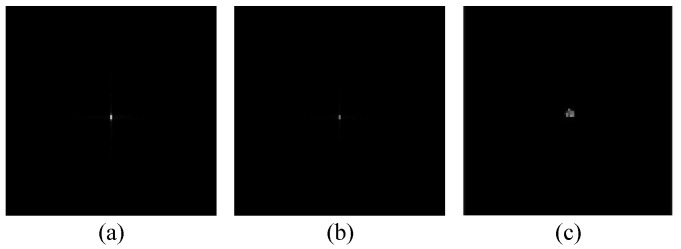
Scan mode moving target imaging: (**a**) image before refocusing; (**b**) image after refocusing; and (**c**) image after deep learning.

Using the above algorithm model, test results from simulations using image data are shown in Figure 4 and Figure 5. For comparison, we also obtained imaging results from deep learning using the U-net convolutional neural network. The up sampling and down sampling use 3 × 3 standard convolution kernels. Adam optimizer is used for learning. Its learning rate is 0.001 and the batch size is 1. Figure 4 and Figure 5 show the correction effect in the before- and after-refocusing images. The Adam optimizer can more accurately display the position of the target in the refocused images.

The image entropy and contrast of image data before and after refocusing are used to evaluate the effect of the proposed algorithm. The image entropy and contrast are calculated using Formulas (12) and (13). In the entropy calculation Formula (12), *pi* represents the gray-level probability in 1D. In 2D, *pi* represents the neighborhood gray-level relationship probability. Formula (13) calculates the contrast, in which *δ*(*i*, *j*) represents the gray-level difference of adjacent pixels, and *P_δ_*(*i*, *j*) represents the probability of occurrence of the differences in gray level.

(12) $$H=-\sum_{i=0}^{255}p_{i}\log p_{i}$$

(13) $$C=\sum_{\delta }\left[\delta {\left(i,j\right)}^{2}P_{\delta }\left(i,j\right)\right]$$

**Figure 6:**
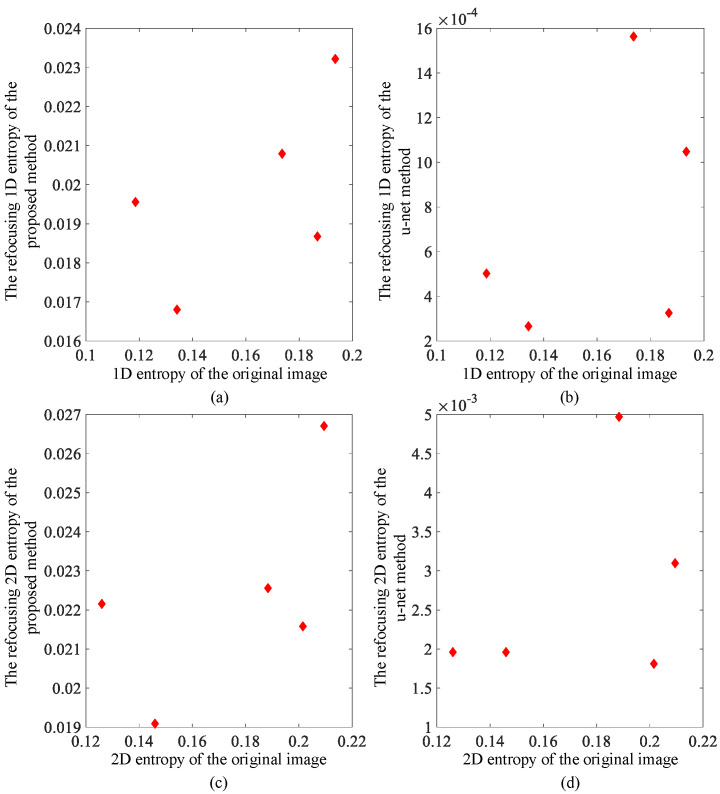
Strip mode image entropy comparison: (**a**) one-dimensional entropy change using proposed method; (**b**) one-dimensional entropy change using deep learning method; (**c**) two-dimensional entropy change using proposed method; and (**d**) two-dimensional entropy change using deep learning method.

**Figure 7:**
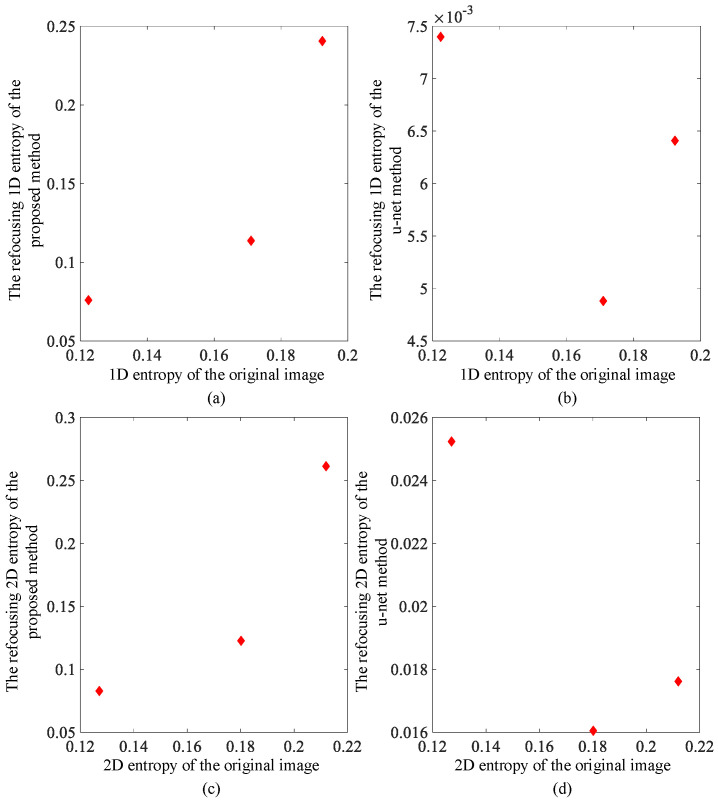
Scan mode image entropy comparison: (**a**) one-dimensional entropy change using proposed method; (**b**) one-dimensional entropy change using deep learning method; (**c**) two-dimensional entropy change using proposed method; and (**d**) two-dimensional entropy change using deep learning method.

Figure 6 and Figure 7 show the one-dimensional and the two-dimensional entropy comparison diagrams before and after refocusing. It can be seen that the image entropy decreases after refocusing. The probability of the corresponding image grayscale value decreases, and the degree of aggregation increases. In the image, it is shown that the grayscale value in the area where the target is located is more aggregated. For comparison, the reduction of entropy value is more obvious after U-net convolutional neural network processing because the maximum value of grayscale is preserved.

**Figure 8:**
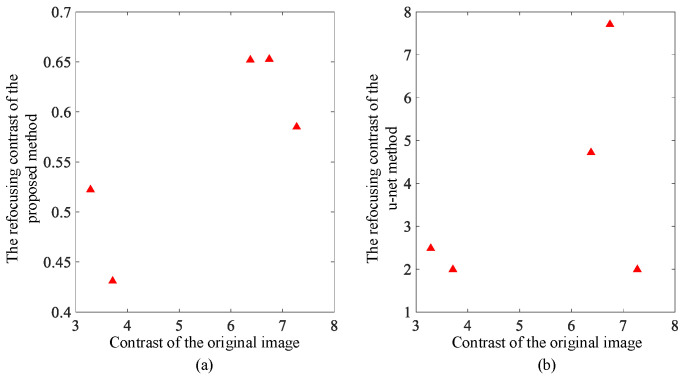
Strip mode image contrast change comparison: (**a**) contrast change with proposed fine focus; and (**b**) contrast change with deep learning method.

**Figure 9:**
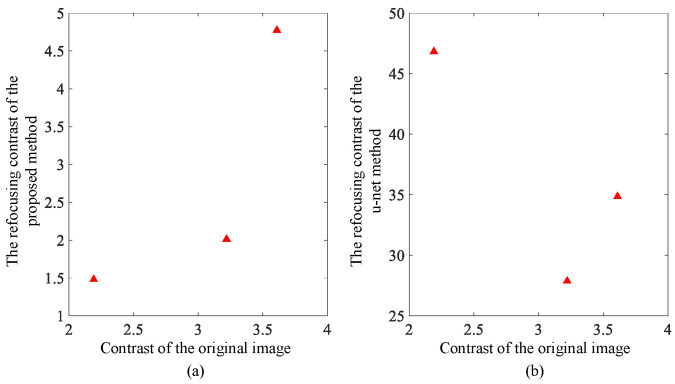
Scan mode image contrast change comparison: (**a**) contrast change with proposed fine focus; and (**b**) contrast change with deep learning method.

The contrast results of refocusing are shown in Figure 8 and Figure 9. The contrast of the images before and after refocusing is significantly reduced in the strip mode. The large residual image of the moving target in the initial image of the strip mode leads to a large grayscale value. The area of the intensity value is relatively wide, which leads to the obvious contrast in the original image. Due to the correction and compensation of the target afterimage after fine focusing, the average grayscale value of the original afterimage area decreases, resulting in a significant reduction in the overall contrast of the image. Relatively speaking, the degree of contrast reduction in the scan mode is much smaller than that in the strip mode due to the small difference between the images before and after. The lateral contrast of the two modes of image contrast results processed by the deep learning method shows an increase in contrast in the scan mode, and the focused imaging results in the corresponding images are not ideal.

**Figure 10:**
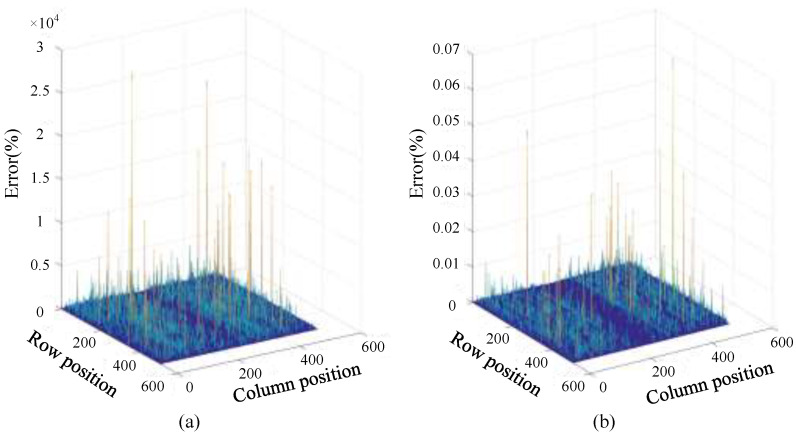
Strip mode relative error: (**a**) single-precision fixed-point and floating-point error; and (**b**) floating-point single-precision and double-precision error.

**Figure 11:**
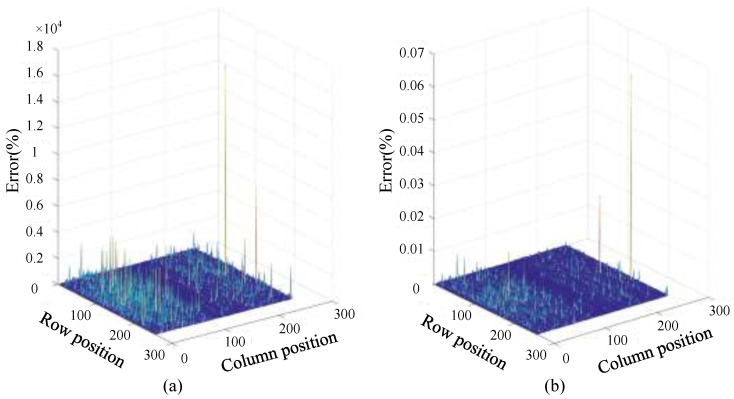
Scan mode relative error: (**a**) single-precision fixed-point and floating-point error; and (**b**) floating-point single-precision and double-precision error.

Data accuracy is a necessary design consideration when implementing algorithms in hardware. Usually, lower data precision can save hardware resources and computing time, but at the same time it can reduce the result precision. For the two modes, the precision and decimal storage mode of the data need to be considered. For the convenience of comparison, the test data are divided into three types: single-precision fixed-point numbers, single-precision floating-point numbers and double-precision floating-point numbers. According to the relative error calculation in the two modes shown in Figure 10 and Figure 11, it can be seen intuitively that in the choice between fixed-point numbers and floating-point numbers, the average value of the relative errors formed by fixed-point numbers has exceeded 100%, indicating that the data calculation is due to the fixed-point numbers. The characteristic of the limit of the number of points causes the data to generate overflow errors in the calculation and cause the calculation result to be wrong. In the relative error comparison between single precision and double precision, it can be seen that the maximum error does not exceed 0.1%. The number of digits required for data storage corresponding to single precision is 32, while that of double precision is 64. For the floating-point calculation IP encapsulated in the development platform, the floating-point calculation IP used by the Fourier correlation transform can calculate the data width. It cannot reach 64 bits, and in order to reduce the amount of data resources, the data type using single-precision floating-point numbers is finally selected for system operation in terms of hardware.

## 3. Refocus System Design

**Figure 12:**
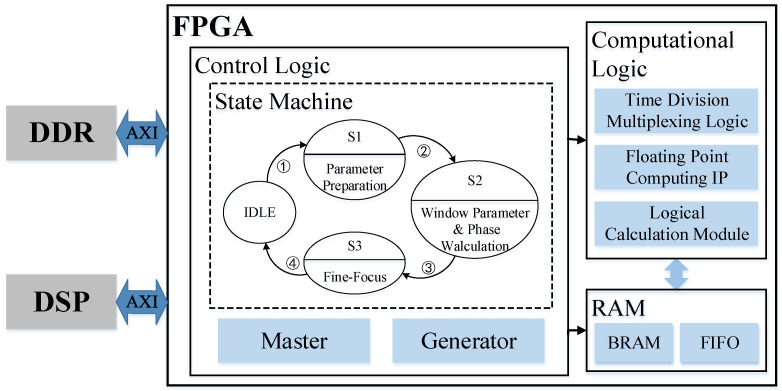
Block diagram of FPGA implementation of the refocusing system.

The proposed FPGA-based SAR imaging refocusing system adopts an “FPGA + DSP + external storage” and uses the slices of the target image to realize the refocusing of moving targets, as shown in Figure 12. The main control logic is responsible for data flow scheduling and module driver utilization for control. Through the identification of the status bits by the control module, the slice data in the DDR is read in the corresponding mode multiple times. Cooperate with calculation logic and RAM storage to realize parameter preparation, compensation phase calculation, and fine focus calculation functions in the algorithm model.

### 3.1. Parameter Preparation State

**Figure 13:**
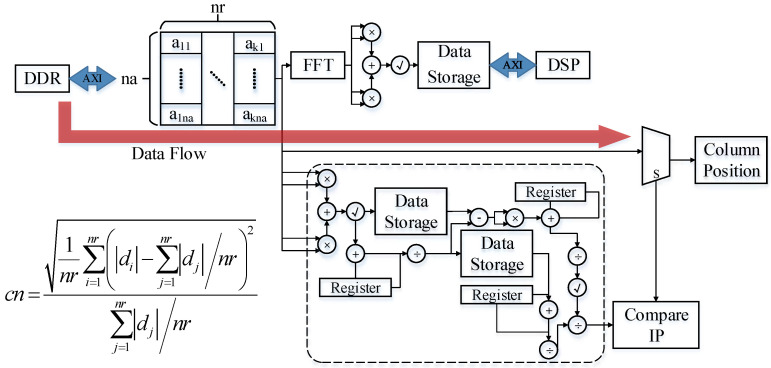
Data flow in the parameter preparation state.

As shown in Figure 13, the parameter preparation state implements two functions in parallel. One function is to sort and filter data for azimuth velocity matching. According to the formula, the coefficient of variation solution module is designed, that is, the modular standard deviation is divided by the modular mean module, which is used to calculate the contrast range azimuth. The sorting part uses the encapsulated floating-point number comparator IP to realize the sorting function through different state jumps of the internal state machine of the sorting module. The transition of the state machine is designed according to the insertion algorithm model. Another function is to perform a Fourier transform on the slice data and realize the calculation of the Doppler center frequency and the calculation of the Taylor window required by the strip mode through DSP, and calculate the corresponding phase data for different moving target speeds for preparation. That is, the complex frequency-domain amplitude calculation after the slice data frequency-domain transformation is completed by FPGA. The calculation results are sent to the DSP according to the AXI protocol, and the complex Doppler center frequency formula calculation is realized through the DSP.

### 3.2. Compensation Phase Calculation State

**Figure 14:**
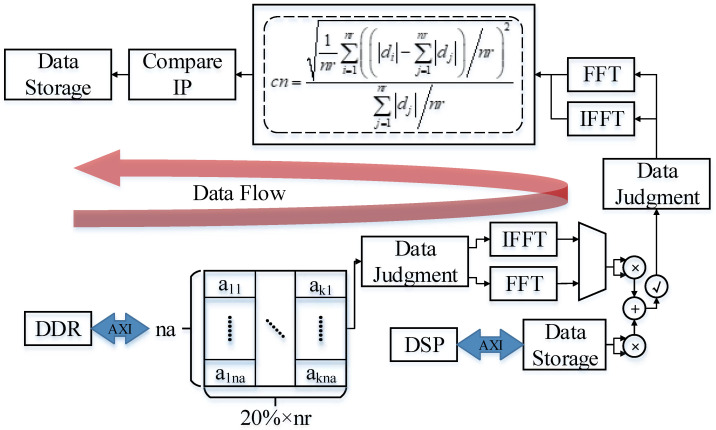
Data flow in the compensation phase calculation state.

As shown in Figure 14, the second reading of slice data is used in the calculation of the compensation phase state. By estimating the azimuth velocity and sending it to the DSP to calculate the compensation phase required for fine focusing. Since the newly defined formula for calculating the contrast range azimuth is the same as the formula used for data sorting and screening in the parameter preparation state, there is only a difference in the input data. Therefore, the coefficient of variation module can be time multiplexed with the one called by the first data stream. The input data of the coefficient of the variation module called in this state is the result of multiplication of the filtered slice data column and the phase data corresponding to the velocity in each azimuth direction calculated by the DSP in the frequency domain. After calculating all the newly defined contrast range azimuth data, a maximum search is performed on them. Take the speed corresponding to the maximum of the newly defined contrast range azimuth, and send it to the DSP to calculate the azimuth, speed, and phase. In the strip mode, the DSP returns the Taylor window to the FPGA for storage at the same time.

### 3.3. Fine Focus State

**Figure 15:**
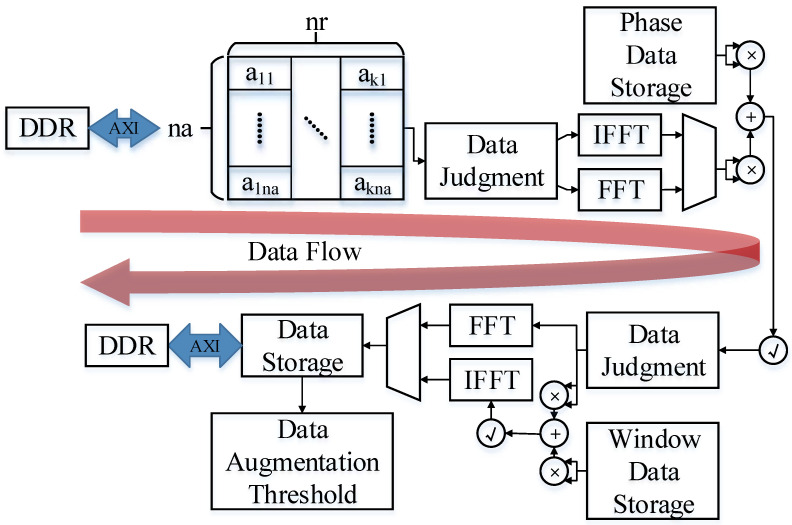
Data flow in the fine focus state.

As shown in Figure 15, in the fine focus state, the fine focus of the moving target is achieved using the calculated compensation phase. Compared with the first two states, the idea of data processing in this state is relatively simple. After the image data are converted into the frequency domain, the complex multiplication operation corresponding to the phase is performed. Select whether to perform windowing processing according to the mode, and then convert the data into the time domain and return it. The required parameters are all sent to FPGA by DSP for storage in the last state. The FPGA only needs to read it at the corresponding position to perform corresponding calculations with the slice data. In this state, complex multiplication is implemented in the same way as the remaining two states. However, due to the consideration of data scheduling complexity and data pipeline design, complex multiplication cannot be used for resource reuse. The final fine focusing result is passed in parallel, and the subsequent data optimization part is passed in and returned to the DDR for storage.

## 4. System Optimization

### 4.1. Fine Focus Result Enhancement and Quantization

The refocusing is performed using an FPGA, with image data of 32-bit floating-point numbers. However, transmitting the refocused image to the next stage creates an imbalance in bandwidth and transmission speed. The refocused image data of SAR imaging need to be sent to the next level for identification and detection. According to the refocusing effect of software, it can be seen that the effect of the moving target after the overall image slice is not outstanding. In this paper, the threshold control enhancement method is used to expand and enhance the row and column data corresponding to the slice where the target is located, highlighting the position and effect of the target. In terms of data compression and quantization, this paper clears the image data other than the moving target. Further, it only enhances and preserves the row and column data of the slice where the target is located to highlight the position and characteristics of the moving target and shorten the data width. The data content of each data point is reduced to an 8-bit-wide fixed-point integer to realize the overall compression and quantization of the data.

The enhancing algorithm first calculates the data characteristics of each column based on the finely focused slice data, including mean, standard deviation, and maximum and minimum values. The minimum value is set as the lower limit *P_min_*, and the degree of dispersion of the single-column data are judged according to the size of the variation coefficient. The reverse adjustment of the upper limit *P_max_* is performed according to the increase of the degree of dispersion. *P_max_* is shown in Formula (14), where the size of *b* is inversely proportional to the degree of dispersion of column data:

(14) $$P_{\max}=d+b\times S$$

The slice data after fine focusing is compared with the threshold. According to the result of data normalization, the grayscale compression of the image is carried out. The highest grayscale level is reached when the data exceed the upper limit. Formula (15) shows how the enhanced data *D* are obtained:

(15) $$D_{i}=\left\{\begin{array}{cc} \left\lfloor 255\times \left(d_{i}-P_{\min}\right)/\left(P_{\max}-P_{\min}\right)\right\rfloor \times T_{\mathrm{mid}} & d_{i}\leq P_{\max}\\ 255+\left\lfloor 255\times \left(d_{i}-P_{\min}\right)/\left(P_{\max}-P_{\min}\right)\right\rfloor \times T_{\mathrm{mid}} & d_{i}>P_{\max} \end{array}\right.$$

where *D_i_* is the *i*-th data in the column of slice data after enhancement, and *d_i_* is the *i*-th in the column of slice data after fine focusing, when *d_i_* ∈ [*P_min_*, *P_max_*], *T_mid_* = 1, otherwise *T_mid_* = 0.

**Figure 16:**
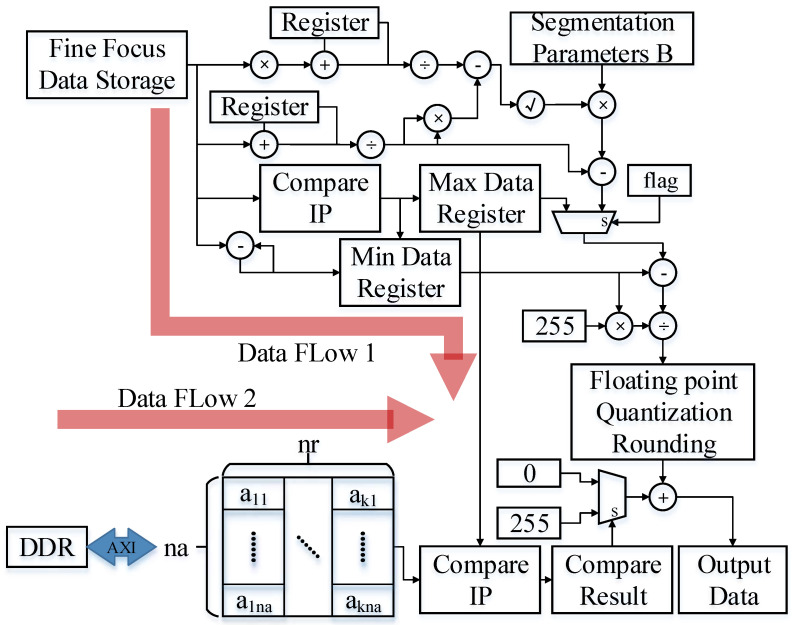
Data flow of data enhancement.

Continuing the design method of algorithm splitting, the parameters required for data enhancement are calculated as two thresholds. The two thresholds need to calculate the data features of the slice data after fine focusing. Corresponding to the hardware design idea, since the obtained threshold needs to be calculated on the whole data of the slice, as shown in Figure 16, the data enhancement is split into two data streams, one for the threshold calculation and another for the final enhancement calculation. In the threshold calculation data flow, the data source can be directly connected to the slice data after fine focusing. While performing threshold calculation on the fine focus result, return it to DDR. In order to correspond to the calculation synchronization, some data features need to be stored with delay. Finally, the threshold is calculated according to the synchronous input of the formula. The slice data after fine focusing is read in for the second time for data enhancement calculation. According to the formula, it is necessary to arrange the logical resources required for calculation according to the calculation sequence. Pay attention to the calculation method conversion between floating-point numbers and fixed-point numbers. When implementing the data enhancement Formula (15), there is no packaged computing IP inside the FPGA for floating-point rounding. Therefore, according to the composition of floating-point numbers, it is necessary to carry out the reverse design of converting floating-point numbers to fixed-point numbers and rounding up.

**Figure 17:**
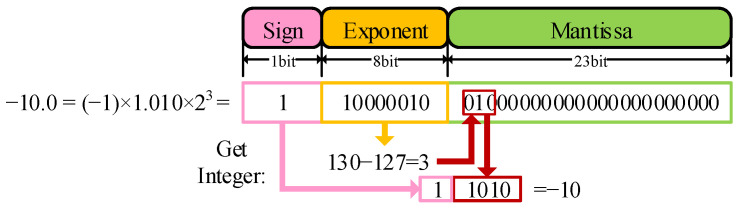
Fixed-point conversion.

As shown in Figure 17, the 32-bit floating-point number is rounded, and it is more complicated to build a fixed-point number generation module in reverse according to the composition of the floating-point number. According to the formula, it is not difficult to see that the essence of this module is rounding calculation. It only needs to take its order code as the judgment condition, intercept the integer part of the data through the mantissa, and then perform a simple calculation on it as the output result.

**Figure 18:**
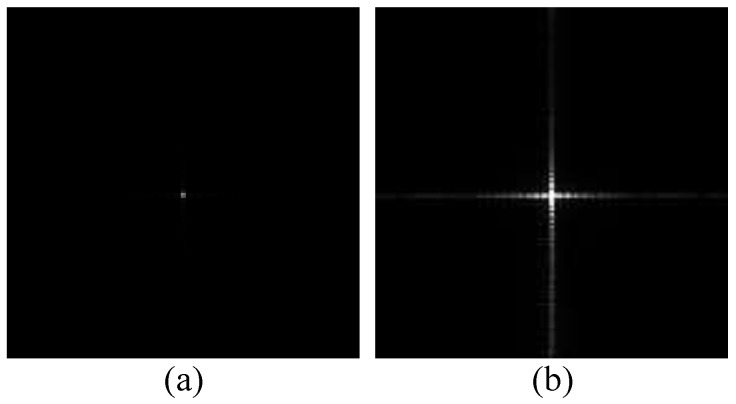
Scan mode data enhancement results: (**a**) before enhancement; and (**b**) after enhancement.

**Figure 19:**
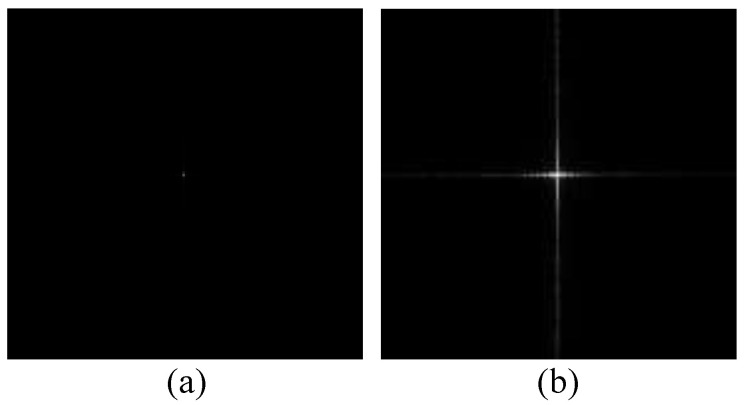
Strip mode data enhancement results: (**a**) before enhancement; and (**b**) after enhancement.

According to the optimized implementation of data enhancement, the focusing results are used for verification, and the overall imaging results are shown in Figure 18 and Figure 19.

### 4.2. Optimization of Speed Estimation Algorithm

**Figure 20:**
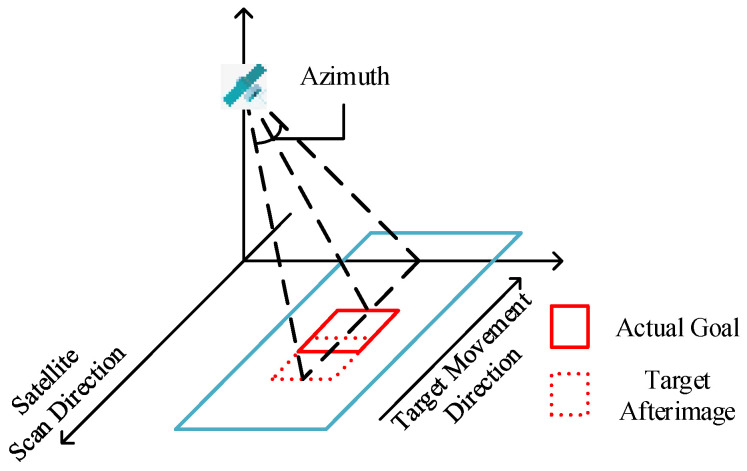
Illustration of SAR speed estimation.

**Figure 21:**
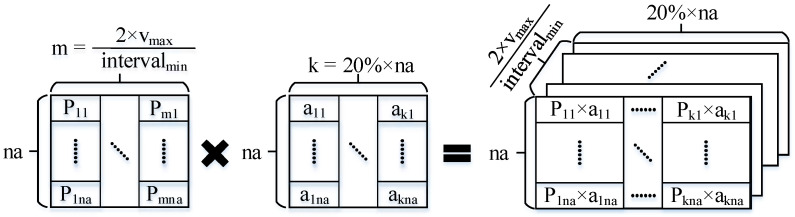
Phase compensation during speed estimation.

In the framework of the SAR imaging refocusing algorithm, the estimation of the azimuth velocity is shown in Figure 20, which is obtained by searching the approach potential according to the maximum velocity of the target. Using the maximum speed of the moving target as the upper limit of speed estimation, the minimum speed interval is defined, and the two-way speed discretization is carried out according to the speed interval. Then according to the Formulas (5) and (6), the phase vector is constructed by using the discretized velocity. Use the phase vector to perform phase correction and compensation on the filtered slice data. The compensated data matrix is constructed as the azimuth *cnr* of the new comparison range, which comprise the data required by Formula (11). The details are shown in Figure 21.

**Figure 22:**
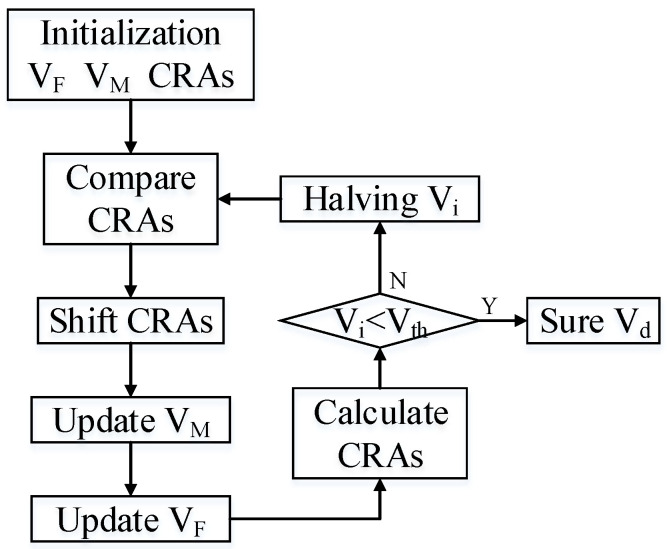
Flowchart of the binary search implemented in the proposed system.

Since the method used is a velocity approximation estimation, it needs to be corrected and selected through comparison. In order to reduce the computational complexity and amount of calculation, this paper optimizes the algorithm for speed estimation to reduce the amount of calculation required. Ordinary comparison methods require a large amount of data to be constructed, and the number of comparisons is fixed according to the degree of data discretization and cannot be optimized. It is necessary to choose a faster comparison method. As a relatively common method in comparison search, the binary search method is optimized in terms of data calculation and comparison. Combining with a binary search method for speed estimation, a fast positioning search is performed by changing the speed interval, and the smallest data interval is used as the judgment condition for the end of the search. The specific calculation method is shown in Figure 22. Among them, *V_M_* is the main speed, which is determined according to the maximum value in CRAs. *V_F_* comprises two slave speeds, which are obtained according to the acceleration and deceleration interval of the main speed. CRAs is the matrix of contrast range azimuth, and its elements are calculated from the corresponding calculation of the main velocity and the slave velocity. *V_i_* is the velocity interval. *V_th_* is the minimum threshold of the speed interval. *V_d_* is the final azimuth velocity to be obtained.

**Figure 23:**
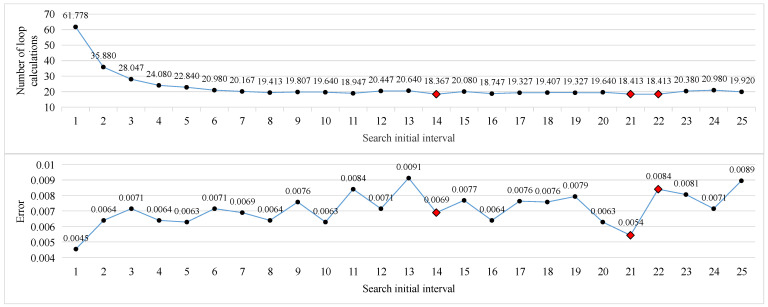
The average number of calculation cycles for each initial interval (**top**), and the average relative error for each initial interval (**bottom**).

**Figure 24:**
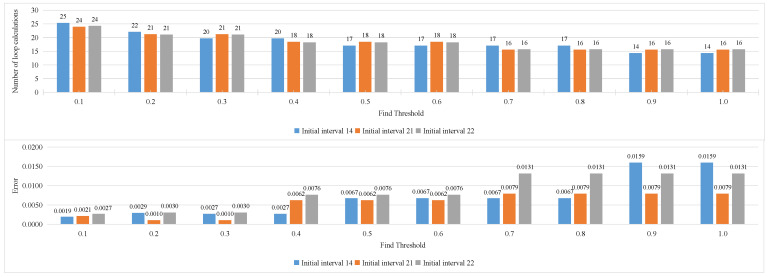
The average number of calculations for each threshold (**top**), and relative error of average cycle for each threshold (**bottom**).

In the binary search method, two more critical parameters need to be limited. Respectively, these are the step size of the initial search interval, and the threshold interval for judging the end of the search. Adjust the initial step size and threshold according to the maximum speed of the moving target. The result of comprehensive training of the two modes using multiple images is shown in Figure 23. It is not difficult to see from the figure that when the initial step size is greater than 7, the number of cycle calculations experienced by the speed estimation tends to be relatively stable, and it is relatively smaller when the initial step size is 14, 21, and 22. Its corresponding calculation error is shown in the line chart below it. For these three initial steps, the threshold value is further analyzed, and the results are shown in Figure 24. It is not difficult to see that the smaller the threshold, the higher the accuracy of speed estimation, but the higher the number of calculations required. Comparing the calculation errors of the three initial step sizes horizontally, it is not difficult to see that the parameter combination with a threshold of 0.3 and an initial step size of 21 has certain advantages.

According to algorithm optimization, the binary search method fundamentally reduces the amount of slice column data through screening, and reduces the number of phase vectors that need to be calculated through the binary search method. The reduction in the number of reads has increased the speed to a certain extent. And because the floating-point number multiplication is the main calculation delay when the FPGA is implemented, the number of multiplications between data is reduced, and a large amount of processing time is saved.

**Figure 25:**
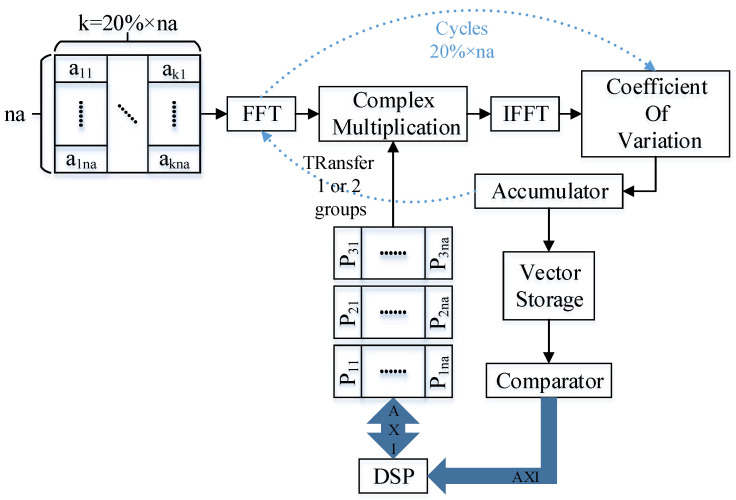
Implementation of speed estimation.

The corresponding FPGA design is shown in Figure 25. When taken together with Figure 19, it can be seen that the binary search method is realized by controlling the AXI bus to read the phase data calculated by the DSP and controlling the number of phases read by comparing the results of the modules. According to the algorithm, the phase data are sent to three groups by DSP through the AXI bus, corresponding to three speeds. According to the calculation cycle of the algorithm, three new contrast range azimuths are obtained, and the corresponding phases are stored. By comparing with the contrast range azimuth after phase compensation, the number of phase vectors to be read is controlled, and corresponding addresses are replaced and stored. Adjust again to enter the calculation loop. The number of azimuth angles and the storage position of the new comparison range obtained in the subsequent calculation cycle are controlled according to the updated number of phase vectors and the storage position. Until the speed interval in the comparison module reaches the minimum threshold, the speed estimation of the binary search method ends. Since the fine focusing uses the phase compensation method, the calculation of the phase is consistent with the phase calculation method of the velocity estimation. In the hardware implementation, there is no need to extract the velocity parameters, and the phase vector is directly stored for subsequent calculations.

### 4.3. Timing Optimization

**Figure 26:**
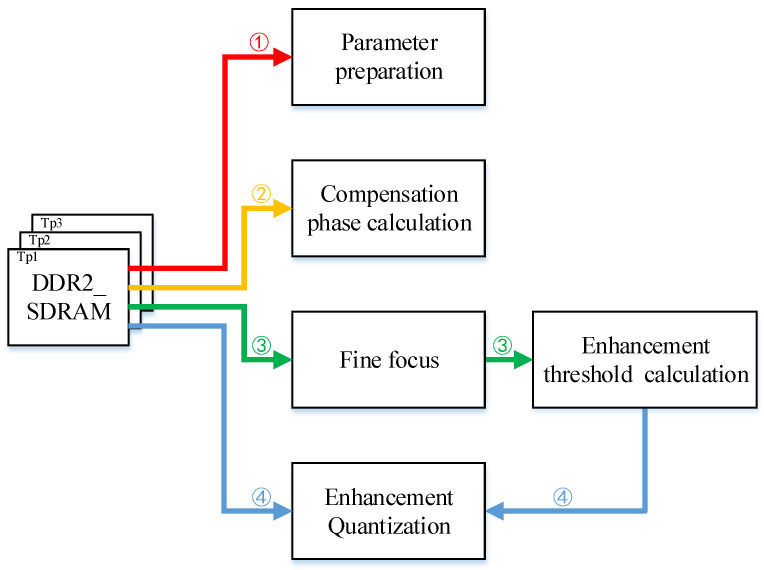
Data flow diagram of the overall system. (1) First reading of the original slice data and enter the parameter preparation state. (2) Second reading and enter the phase compensation state. (3) Third reading and enter the fine focus state. After a column of data is imported and calculated in fine focus, the results are transferred to the enhancement threshold calculation state. (4) Fourth reading and enter the enhancement quantization state.

After adding the enhancement and quantization links in the previous section, the refocusing system will change from three states to four states. They are parameter preparation, compensation phase calculation, fine focusing, and threshold and enhancement calculation. According to the data flow rules, each part of the data driver needs to read the corresponding slice data from the DDR SDRAM for processing. However, the data threshold calculation is a preparatory work for the data enhancement calculation, and the data driver used to realize this part of the logic does not need to wait for the complete fine focus image result. After fine focusing and processing the data of each column, the data can be stored in parallel and sent to the enhanced threshold calculation part to start logic implementation. The calculation of the enhanced threshold is completed while achieving the fine focus of the entire slice. This approach reduces data interaction and improves computational efficiency. The specific state is shown in Figure 26, where the sequence of the data flow is labeled. 

Combined with the overall data flow in Figure 26, the data processing logic can be divided into refocusing implementation and data enhancement quantization implementation according to the data-driven type. The input data required in refocusing are slice raw data. The data required for the realization of the data enhancement and quantization part are all fine focused data. We denote the realization time of single-slice refocusing as *T_rf_*, the time of data enhancement quantization as *T_eq_*, the time of processing a slice as *T_p_*, and the time of processing the whole image as *T_sum_*. According to Formula (16), the processing time of a slice can be obtained, and according to Formula (17), the processing time of a complete imaging picture can be obtained.

(16) $$T_{p}=T_{rf}+T_{eq}$$

(17) $$T_{sum}=\sum_{i=1}^{n}T_{pi}=\sum_{i=1}^{n}\left(T_{rfi}+n\times T_{eq}\right)$$

**Figure 27:**
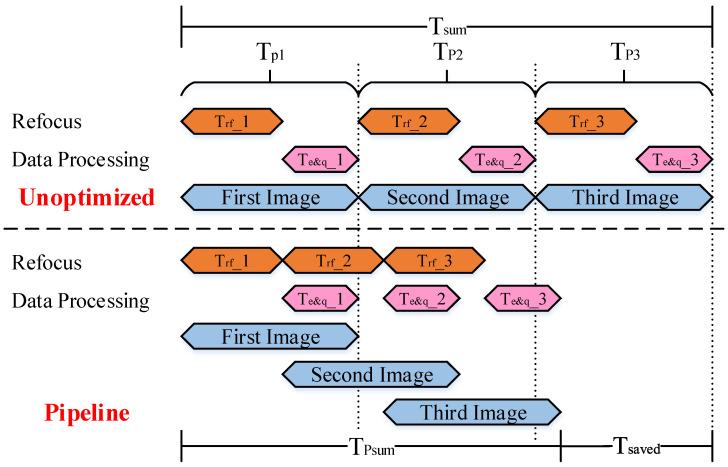
Pipeline optimization of refocusing and data enhancement and quantization.

Assuming that we divide a picture into three data slices, Figure 27 shows the calculation time comparison between the general sequential implementation method and the optimized pipeline structure. It can be found that using the pipeline method, optimization can significantly reduce the calculation time. According to Table 1, which shows the timing comparison of each data flow, it can be seen that hardware processing has an advantage in computing speed. Implementing the pipeline operation in Figure 27, the data enhancement and quantization processing time after refocusing can also be optimized for all remaining slices except the last one. Therefore, the overall executing time is greatly reduced.

## 5. System Verification

**Figure 28:**
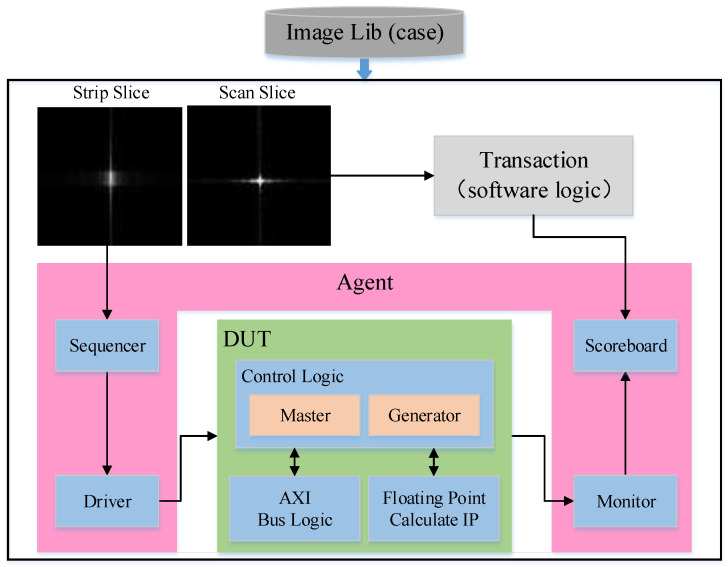
Architecture of the simulation verification system.

To test the hardware design function in accordance with the algorithm model and to simulate the data interaction between the host computer and the FPGA, a verification process was conducted. The overall functional simulation structure diagram is displayed in Figure 28, which simulates the construction method of the UVM verification platform. The verification process uses the original image data of both modes as the required case library. The sequencer and driver modules instantiated in the agent generate stimuli and send them to the DUT for logic reproduction. A monitor is used as a print record module for dynamic storage of output data. While reproducing the DUT, the image data are refocused through software logic. The instantiated scoreboard module is used to compare the data stored dynamically in the monitor with the software output results.

For the FPGA board, Xilinx’s Virtex-5 series XQ5VFX130T-1EF1738I is used. TI’s SMV320C6727 is used for DSP computation. The overall system is realized through the calculation of complex formulas by DSP and the main control logic of FPGA. The internal data interaction is uniformly transmitted according to the AXI interface protocol. The system as a whole performs data sending and receiving judgments according to the handshake method. The overall data flow matches the state of the internal state machine of the main control logic.

Table 2 lists the resource consumption of the hardware design. The main resources consumed are LUT, REG, RAM, and DSP. Due to the requirements of the algorithm, the points of the Fourier transform and the inverse transform of a large amount of data are different, resulting in the utilization of DSP computing resources reaching 90%. The average usage of overall design logic resources exceeds 50%, and the on-chip resources are basically fully utilized and developed.

According to the usage of FPGA resources and referring to other projects, the current power consumption is estimated to be 10 W.

**Figure 29:**
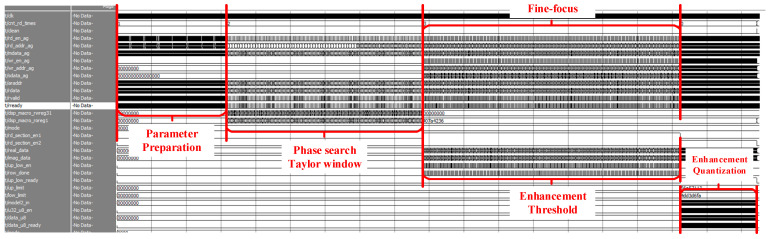
Timing diagram of the complete simulation of refocusing and data processing.

**Figure 30:**
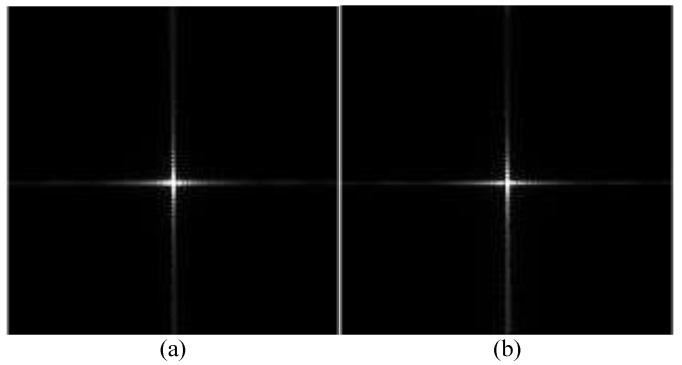
(**a**) Software refocusing imaging results; and (**b**) hardware-in-the-loop (HIL) refocusing imaging results.

Through timing simulation, we can verify the overall function and efficiency of the system. Figure 29 shows the processing time of a single original SAR image. The corresponding modules running status can be found out from the data flow diagram in Figure 26. The results of the prototype verification are shown in Figure 30. In the verification system, the time for the FPGA to process a slice is about 16.236 ms. On the same test set, a computer equipped with a general CPU takes about 3,991 ms to complete a slice processing using MATLAB. Based on the FPGA design, the refocus processing speed is more than 200 times faster than that of general-purpose processors.

## 6. Conclusions

In this paper, we present a Doppler centroid tracking–based refocusing algorithm, which leverages azimuth velocity matching for improved focus. We also conduct hardware implementation and optimization. Through the logical design and acceleration of hardware, the computational efficiency of the refocusing algorithm has been improved. The use of pipelining and serial-to-parallel conversion further reduces the execution time of the algorithm on the FPGA. Resource optimization through module reuse allows us to achieve the refocusing function using limited resources. Additionally, the data enhancement and quantization module improves the target recognition rate and reduces transmission time loss. Experiments show that compared to the software algorithm, our hardware design has achieved a computational capability of two frames per second. We also simplify the system by using an “FPGA + DSP + external storage” design. This paper provides a practical method for design optimization for hardware implementation of refocusing algorithms.

Future work should aim to optimize the resources of the refocusing module and the data quantization enhancement module. Efforts should be made to make maximum use of modules to reduce system resources. Additionally, research should be done to improve the system’s adaptability to different slice data parameter settings by enhancing its reusability.

## Figures and Tables

**Table 1:** The time overhead of states of the refocusing algorithm.

State	Description	Software Calculation Time/s	Hardware Calculation Time/ms	Speed Gain
1	Parameter preparation	1.1370	3.173480	358
2	Speed estimation	0.3020	5.638580	53
3	Fine focus	1.1210	7.424190	151
4	Enhancement and quantization	1.4310	2.206870	648

**Table 2:** System resource utilization.

Resource	Used	XQ5VFX130T Total Resource	Utilization
LUT	46,211	81,920	55%
REG	39,359	81,920	47%
RAM	28	298	9%
DSP	292	320	90%

## Data Availability

Data sharing not applicable. No new data were created or analyzed in this study. Data sharing is not applicable to this article.
